# In memoriam: Stuart J. Connolly, MD, 9 April 1949–2 June 2024

**DOI:** 10.1093/europace/euae184

**Published:** 2024-07-01

**Authors:** Stefan H Hohnloser, Alexander P Benz, Carlos A Morillo, Jeff S Healey, A John Camm

**Affiliations:** Department of Cardiology, J. W. Goethe University, Theodor-Stern-Kai 7, 60596 Frankfurt, Germany; Population Health Research Institute, McMaster University, Hamilton, Ontario, Canada; Department of Cardiology, University Medical Center Mainz, Johannes Gutenberg-University, Mainz, Germany; Department of Cardiac Sciences, University of Calgary, Calgary, Alberta, Canada; Population Health Research Institute, McMaster University, Hamilton, Ontario, Canada; Department of Cardiology, Cardiology Clinical Academic Group, Molecular & Clinical Sciences Institute, St. George’s University of London, London, UK

**Figure:**
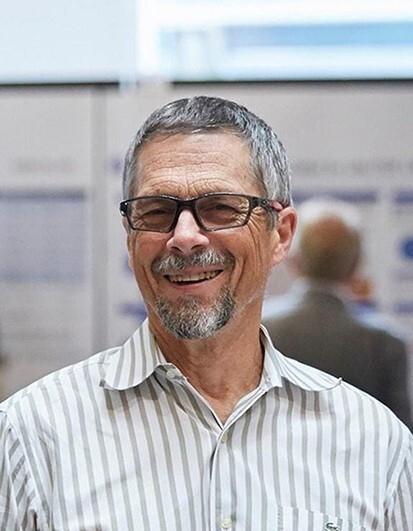


On 2 June 2024, our community lost one of its most prominent researchers and physicians. With dignity and grace, and surrounded by his family, Dr Stuart James Connolly died of neuroendocrine cancer. He was predeceased by his loving wife, Dr Elaine Gordon, and survived by his four children, Katherine, Joseph, Benjamin, and Adam, four grandchildren, and by his partner, Susan Block.

Stuart was a deeply spiritual person, and his honesty and integrity were obvious to everyone who had the good fortune to get to know him. Born and raised in Montréal, he studied philosophy at McGill University and later completed a Master of Philosophy at Fordham University. He then went to medical school at McGill University, and completed his cardiology training at St. Michael’s Hospital in Toronto. Along with Elaine, he then went to Stanford for a fellowship in clinical pharmacology with Roger Winkle. Back in Canada, Stuart became faculty member at McMaster University in Hamilton. During his four decades at this institution, Stuart was instrumental in building the local cardiac arrhythmia service. He later served as the cardiology division director. During his entire career, Stuart was a dedicated mentor to young clinical scientists at McMaster University and beyond.

Stuart’s body of work covers all essential aspects of clinical electrophysiology. He started out with projects such as the CAMIAT trial, led by John Cairns. This study looked at the effects of amiodarone vs. placebo in patients with ventricular ectopy. This Canadian trial was paralleled by a similar European trial, the EMIAT study. Subsequently, Stuart applied the principle of the randomized clinical trial to various fields of electrophysiology, such as cardiac pacing (CTOPP) or implantable cardioverter-defibrillator therapy (CIDS trial). Early on in his career, Stuart started to set up an international network of physicians treating patients with cardiac arrhythmias. This paved the way for many important studies that were carried out simultaneously in Europe and in North America. Many times, Stuart was the critical thinker who started asking questions for which at the time there were no answers. By doing so, Stuart brought the large clinical trial to arrhythmia medicine for the benefit of patients. A good example for a trial with a profound impact is the DINAMIT trial. This trial randomized infarct survivors to receive an implantable cardioverter-defibrillator in addition to usual vs. usual care alone. Against conventional wisdom, the trial failed to show benefit of the ICD within the first weeks after myocardial infarction. Other earlier work was concerned with pharmacological therapy of arrhythmias, such as the OPTIC trial that showed that amiodarone significantly reduced the number of shocks in patients with an ICD, thereby improving quality of life of patients. Another antiarrhythmic drug trial was ATHENA that is the largest trial of its kind comprising 4628 patients with paroxysmal or persistent atrial fibrillation. Along with the subsequent PALLAS trial in patients with permanent atrial fibrillation, these two trials helped define the role of dronedarone, a then novel class III antiarrhythmic drug.

For more than three decades, Stuart’s main mission was to improve stroke prevention in patients with atrial fibrillation globally. In 2018, Stuart held the ESC Geoffrey Rose Lecture on Population Sciences that provided an impressive overview of his endeavours. It all started with the Canadian Atrial Fibrillation Anticoagulation (CAFA) Study published in 1991, one of six pivotal randomized trials that established the important role of vitamin K antagonists for stroke prevention. About a decade later, the ACTIVE trials sought out to identify alternative preventative treatments for patients with atrial fibrillation. Co-ordinated by the Population Health Research Institute/McMaster University in Hamilton, these trials mainly recruited participants from centres located in Europe. ACTIVE W demonstrated the superiority of warfarin over dual antiplatelet therapy for the prevention of stroke, myocardial infarction, systemic embolism, or vascular death. ACTIVE A (64% of study participants enrolled in Europe) showed that dual antiplatelet therapy was superior to aspirin monotherapy in patients who were deemed unsuitable for oral anticoagulation with warfarin. Only shortly thereafter, the global RE-LY trial (37% European participants) reported in 2009 established the direct thrombin inhibitor, dabigatran, ushering in the era of the direct oral anticoagulants and revolutionizing stroke prevention in patients with atrial fibrillation. In 2011, the AVERROES trial demonstrated the superiority of the factor Xa inhibitor, apixaban, over aspirin for stroke prevention in patients unsuitable for oral anticoagulation with a vitamin K antagonist. About another decade later, the INVICTUS trial showed the superiority of vitamin K antagonist therapy over rivaroxaban in patients with atrial fibrillation secondary to rheumatic heart disease, recruiting participants from centres in Africa, South East Asia, and Latin America.

Published in 2012, ASSERT provided a unique opportunity to link Stuart’s interest in patients receiving a pacemaker or ICD with stroke risk assessment. This trial helped define a new clinical entity that is now widely known as device-detected atrial fibrillation. Being testament to Stuart’s persistence and curiosity, it took another 11 years to plan and complete the ARTESiA trial, demonstrating the superiority of apixaban over aspirin for the prevention of stroke or systemic embolism in patients similar to those enrolled in ASSERT. Stuart was also open to other clinical fields and eager to collaborate, which helped create high-quality evidence for treatments tested in patients with embolic stroke of undetermined source (NAVIGATE ESUS), dual pathway inhibition in patients with stable vascular disease (COMPASS), surgical occlusion of the left atrial appendage (LAAOS III), and specific reversal of anticoagulation in patients with factor Xa inhibitor-associated bleeding (ANNEXA-4 and ANNEXA-I).

Many of the randomized trials Stuart pursued changed clinical practice. This is reflected by the fact that most these trials—if not all—were incorporated into practice guidelines on both sides of the Atlantic Ocean. His scientific achievements are tremendous as is his list of publications, comprising more than 500 original articles. Among these are more than 20 manuscripts published in the *New England Journal of Medicine*. Because of its high quality and relevance, his work will continue to impact both patients and physicians across the globe for many years to come.

Apart from work, Stuart had many hobbies such as literature, theatre, jazz music, wine, ballet, making maple syrup, the Toronto Raptors, gardening, and sports activities, in particular skiing and snowboarding. He loved to bring his family and friends out to Muskoka and British Columbia. He picked up road biking in his late fifties and later turned to triathlon racing. As with his clinical and scientific work, only excellence was what counted for him. This spirit placed him second at the 70.3 Ironman World Championship in 2022.

Stuart Connolly will be remembered as an outstanding clinical scientist who brought the randomized clinical trial to the field of electrophysiology and cardiac arrhythmia, as a caring husband, a loving father, an outstanding mentor, and a great friend.

